# Hypoxia inducible prolyl hydroxylase PHD3 maintains carcinoma cell growth by decreasing the stability of p27

**DOI:** 10.1186/s12943-015-0410-5

**Published:** 2015-07-30

**Authors:** Heidi Högel, Petra Miikkulainen, Lucia Bino, Panu M. Jaakkola

**Affiliations:** Turku Centre for Biotechnology, University of Turku and Åbo Akademi University, Tykistökatu 6B, 20520 Turku, Finland; Department of Medical Biochemistry, Faculty of Medicine, University of Turku, Kiinamyllynkatu 10, 20520 Turku, Finland; Department of Oncology and Radiotherapy, Turku University Hospital, Hämeentie 11, 20520 Turku, Finland; Present address: Institute of Biophysics, The Academy of Sciences of the Czech Republic, Brno, Czech Republic

**Keywords:** CDKN1B, Cell cycle, EGLN, HIF, Hypoxia, VHL

## Abstract

**Background:**

Hypoxia can halt cell cycle progression of several cell types at the G1/S interface. The arrest needs to be overcome by cancer cells. We have previously shown that the hypoxia-inducible cellular oxygen sensor PHD3/EGLN3 enhances hypoxic cell cycle entry at the G1/S boundary.

**Methods:**

We used PHD3 knockdown by siRNA and shRNA in HeLa and 786–0 renal cancer cells. Flow cytometry with cell synchronization was used to study cell growth at different cell cycle phases. Total and phosphospecific antibodies together with cycloheximide chase were used to study p27/CDKN1B expression and fractionations for subcellular protein localization.

**Results:**

Here we show that PHD3 enhances cell cycle by decreasing the expression of the CDK inhibitor p27/CDKN1B. PHD3 reduction led to increased p27 expression under hypoxia or VHL mutation. p27 was both required and sufficient for the PHD3 knockdown induced cell cycle block. PHD3 knockdown did not affect p27 transcription and the effect was HIF-independent. In contrast, PHD3 depletion increased the p27 half-life from G0 to S-phase. PHD3 depletion led to an increase in p27 phosphorylation at serine 10 without affecting threonine phosphorylation. Intact serine 10 was required for normal hypoxic and PHD3-mediated degradation of p27.

**Conclusions:**

The data demonstrates that PHD3 can drive cell cycle entry at the G1/S transition through decreasing the half-life of p27 that occurs by attenuating p27S10 phosphorylation.

**Electronic supplementary material:**

The online version of this article (doi:10.1186/s12943-015-0410-5) contains supplementary material, which is available to authorized users.

## Background

Hypoxia is a common feature of solid tumors inducing a number of survival responses such as angiogenesis, induction of glycolytic metabolism and genetic instability. It is also a strong driving force in the clonal selection that supports more aggressive disease (reviewed in [1]). Under hypoxia in many non-transformed cell types the cell cycle is arrested at the G1/S interface [2]. In contrast to the non-transformed cells, cancer cells are often able to exceed the restriction point and to overcome the cell cycle arrest in order to sustain proliferation in hypoxic environment.

Cell cycle arrest in G1 is mediated by reduced activity of G1/S-specific cyclin-CDK complexes and increased expression of cyclin-dependent inhibitors (CKIs) including p27/CDKN1B [3, 4]. Hypoxia has been reported to upregulate several cell cycle inhibitors, including p21(CDKN1A) but the hypoxic cell cycle arrest does not require p21 [5, 6]. Also p16(INK4a) and p27(CDKN1B) are upregulated by hypoxia but their requirement in the hypoxic cell cycle arrest is controversial [5–7]. The expression of p27 is dependent on the cell cycle phase and subcellular localization and it is regulated mainly post-translationally by the ubiquitin-proteasome system through at least two distinct pathways (reviewed in [8]). One of the ubiquitination reactions is initiated by phosphorylation of threonine 187 (T187) by cyclin E/Cdk2 complex in proliferating cells [9, 10]. p27 phosphorylated on T187 is targeted to degradation by SCF^Skp2^ ubiquitin ligase complex that allows cells to enter S phase [11–13]. Moreover, in resting quiescent cells, phosphorylation of serine 10 (S10) markedly increases p27 stability [14, 15]. At cell cycle re-entry S10 phosphorylation also serves as an export signal from nucleus to cytoplasm [16, 17] where the degradation is directed by KPC ubiquitin ligase complex [18, 19]. In addition to the stabilization of p27 in quiescent cells, S10-dependent increase in p27 half-life has been shown to take place at early G1 [20].

Many of the responses to hypoxia are mediated by hypoxia-inducible transcription factor (HIF) that is rapidly degraded in normoxia but stabilized under hypoxia. The activity of HIF is regulated by the stability of its α-subunit, which is hydroxylated by oxygen-dependent prolyl hydroxylase enzymes, the PHDs. Hydroxylation leads to von Hippel-Lindau protein (pVHL) mediated HIF-α degradation. Hence the lack of oxygen or pVHL inactivation causes activation of HIF and downstream pathways (reviewed in [21, 22]). In mammals three prolyl hydroxylase isoforms termed PHD1, PHD2 and PHD3 (also called EGLN2, EGLN, EGLN3, respectively) have been characterized. Despite the similarities of PHD isoforms, several differences in their function and characteristics exist [22]. PHD3 has been shown to be critical for regulation of cellular survival mechanisms [23–26]. PHD3 is also the isoform that shows most robust induction under hypoxia [27–30] and is kept inactive in normoxia by an autophagy regulating protein p62/SQSTM1 [31]. The elevated expression in hypoxia is compensated for the reduced activity under hypoxia and PHD3 is known to retain much of its enzymatic activity under hypoxia [32, 33]. Noticeably, PHD3 has been suggested to have the widest range of non-HIF targets and downstream effectors [34].

Out of the three PHDs, PHD1 and −3 have been reported to regulate cell cycle [24, 35]. PHD1 depletion arrests cells under normoxia at G2 as PHD1 causes proline hydroxylation and degradation of the centrosome component Cep192 [35]. We have previously shown that the cellular oxygen sensor PHD3 is required for carcinoma cell cycle progression under hypoxia. PHD3 depletion caused reduced hypoxic carcinoma cell survival, hypophosphorylation of pRb and cell cycle arrest at G1. Concomitantly, PHD3 knockdown induced the expression of p27 in hypoxic conditions without affecting other hypoxia inducible CKIs p16 or p21 [24]. Here we show that PHD3 maintains carcinoma cell growth and enhances cell cycle progression by decreasing the stability of p27. PHD3 depletion increases the expression of p27 phosphorylated at serine 10, a stabile p27 form, leading to high p27 level. Our data argues that PHD3 enhances carcinoma cell cycle through decreased p27 protein level.

## Results

### PHD3 depletion induces p27-mediated cell cycle block

**Fig. 1:**
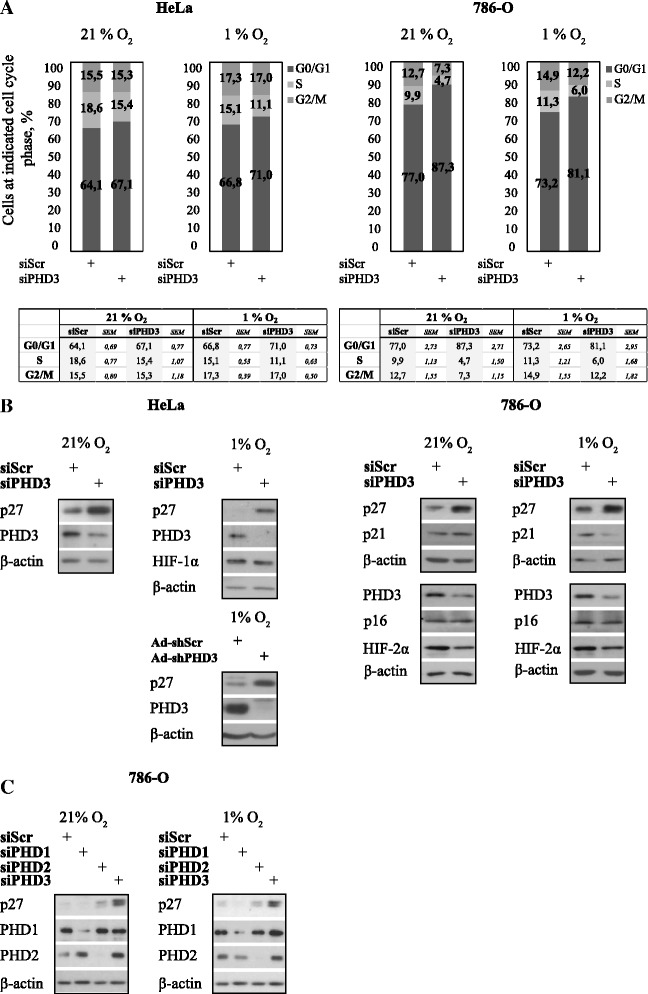
Cell cycle block under PHD3 depletion is accompanied by p27 induction. **a** PHD3 depletion induces a cell cycle block in G0/G1. HeLa and renal cell adenocarcinoma cells (786-O) were transfected with control (siScr) or PHD3 targeted (siPHD3) siRNA followed by synchronization at G0 and 24-h hypoxic exposure. Cell cycle progression was monitored by FACS analysis 8 h after cell cycle release. The combined means of three independent experiments are presented (±SEM) shown in the tables below. **b** PHD3 depletion induces p27 expression in HeLa cells and in 786-O cells under hypoxia (1 % O_2_) and normoxia (21 % O_2_) by siPHD3 and independent adenoviral shRNA against PHD3. p21 or p16 expression is not elevated by PHD3 knockdown. **c** Depletion of either PHD1 or PHD2 by siRNA does not increase p27 expression in 786-O cells

We have previously shown that depleting PHD3 induces cell cycle arrest in carcinoma cell lines (HeLa and SCC cells) at G1 that is mainly seen in hypoxic (1 % O_2_) conditions with elevated PHD3 [24]. We now further analyzed the PHD3-depletion induced cell cycle arrest using synchronized HeLa cells and renal cell adenocarcinoma cells (786-O), the latter bearing VHL inactivation and high basal PHD3 expression. For PHD3-depletion well-characterized siRNA was used [24, 31, 36, 37]. We have previously further validated the siRNA in PHD3-mediated cell cycle regulation using point mutated siRNA and rescue experiments as control [24]. In line with our previous studies using non-synchronous cells, the synchronized HeLa cells show markedly reduced cell subpopulation at the S-phase under PHD3 depletion (siPHD3) at eight hours after release from synchronization as compared to control. Accordingly, cells in G1 phase were increased by PHD3 depletion (Fig. 1a). In 786-O cells the growth arrest was even more pronounced both in hypoxic (1 % O_2_) as well as in normoxic cells (21 % O_2_) the latter showing as high PHD3 expression as the hypoxic cells (Fig. 1a and b). Moreover, independent adenoviral shRNA and plasmid-delivered shRNAs showed similar PHD3-dependent inhibition in hypoxic cell growth (Additional file 1: Figure S1).

In line with our previous studies using SCC cells, HeLa cell showed a PHD3-dependent induction in p27 expression under hypoxia when using either siRNA or an independent adenoviral shRNA against PHD3 (Fig. 1b). Likewise, elevated p27 expression was seen in 786-O cells both under normoxia and hypoxia. Noticeably, as shown previously in HeLa cells neither p21 nor p16 CKIs were affected by PHD3-depletion confirming the p27-specific effect of PHD3 (Fig. 1b) [24]. The hypoxic increase in p27 expression seems to be specific for PHD3 as neither PHD1 nor PHD2 depletion had major effect on p27 expression (Fig. 1c). In fact, PHD1 depletion slightly decreased p27 expression, which might be due to PHD1-induced changes in cell cycle distribution caused by defect in centrosome duplication [35].

**Fig. 2:**
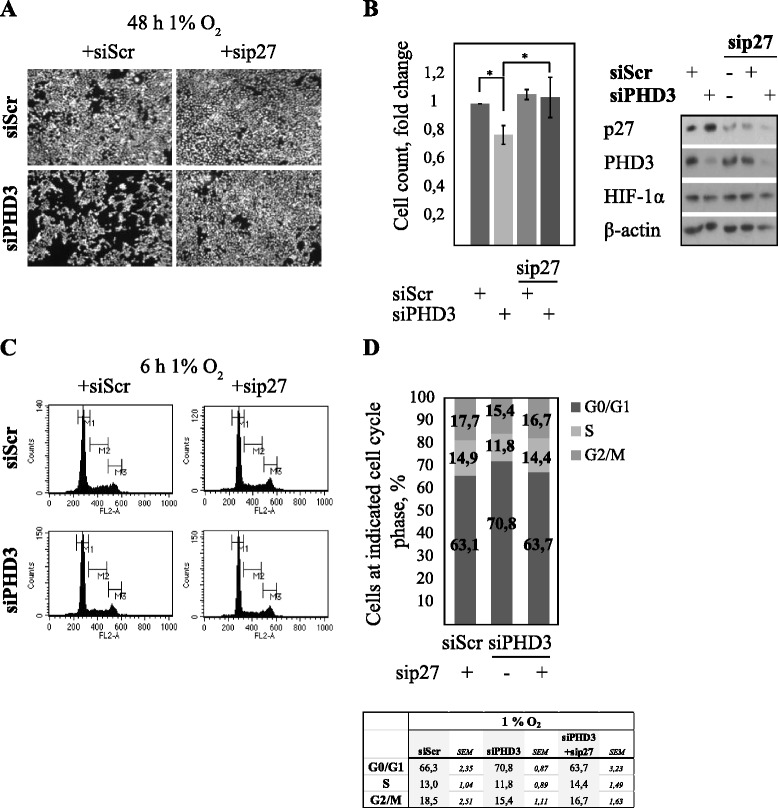
Cell cycle block in PHD3 depleted cells is p27-dependent. **a** Reduced cell proliferation as judged by reduced cell amount of hypoxic siPHD3-depleted HeLa cells is rescued with concomitant p27 knockdown. **b** Quantification of cell survival from four independent experiments with 4–5 optical views per experiment. Asterisk indicates significant difference (*p* = 0,05; *n* = 4). Protein expression was monitored with western blotting from parallel samples. **c** Knockdown of p27 restores the effect of PHD3 depletion on cell cycle. HeLa cells exposed to the indicated double knockdown followed by 6 h hypoxia and FACS analysis. **d** Quantification of cell cycle phases from three independent experiments using indicated double knockdown

To study the role of p27 in siPHD3-mediated cell cycle block we used PHD3/p27 double knockdown followed by cell amount analysis in hypoxia. The reduced cell amount under hypoxia by PHD3 depletion was reverted by simultaneous p27 knockdown (sip27) (Fig. 2a). Quantification of cell amount showed nearly complete restoration of PHD3-induced growth arrest by sip27 (Fig. 2b). We further performed cell cycle analysis using PHD3/p27 double knockdown. In line with cell growth, the inactivation of p27 rescued siPHD3-induced block at G1 (Fig. 2c and d). The data demonstrated that p27 is both necessary and sufficient for siPHD3-induced cell cycle block at G1 and that p27 has a direct role downstream of PHD3 in cell cycle regulation.

### PHD3 depletion induces HIF-independent post-transcriptional p27 expression

**Fig. 3:**
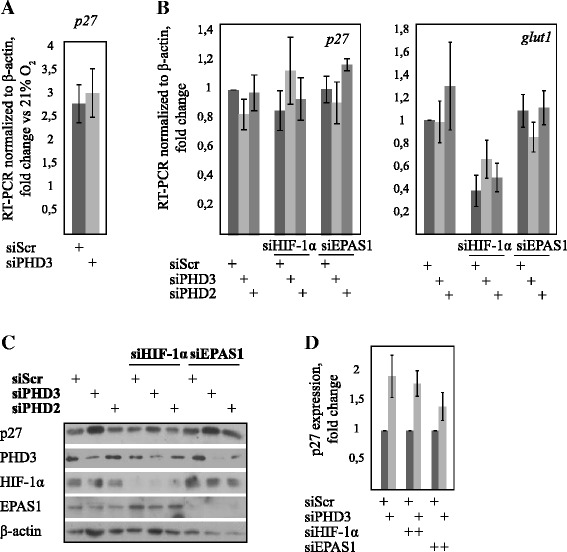
PHD3 elevates p27 expression through a post-translational mechanism. **a** PHD3 depletion has no effect on p27 transcription under hypoxia. p27 mRNA levels were measured in HeLa cells using quantitative real-time PCR. Results shown as fold change vs normoxic control, four independent experiments (± SEM) (*p* = n.s.; *n* = 4). **b** Hypoxic p27 expression is HIF-1α and EPAS1/HIF-2α independent. QRT-PCR analysis of p27 and hypoxia-inducible *glut-1* mRNA normalized to *β-actin* using the indicated double knockdown after 24 h of hypoxia. Unlike *glut-1* HIF knockdown has little effect on p27 transcription. Results from three independent experiments (±SEM) are shown (*p* = n.s.; *n* = 3). **c** PHD3 depletion induces p27 protein levels independently of HIF-1α or EPAS1/HIF-2α depletion in HeLa cells. **d** Quantification of p27 protein expression using indicated double knockdown. Results from three independent experiments (±SEM) are shown

We next asked whether PHD3 affects p27 expression at the mRNA level and whether the induction is HIF-dependent using Q-RT-PCR in siPHD3 transfected cells both in normoxic and hypoxic conditions. As published previously, hypoxia induced p27 transcription almost threefold from normoxic level by an HIF-independent mechanism [6]. However, once under hypoxia there was no significant difference on p27 mRNA level between control and PHD3 depleted cells (Fig. 3a). This was further confirmed by studying HIF dependency of p27 under hypoxia in HeLa cells. Double knockdown with siPHD3 together with either HIF-1α or EPAS1/HIF-2α targeted siRNA had no significant effect on p27 mRNA in hypoxia whereas the mRNA of a well-characterized HIF-1 target *glut1* was markedly reduced as expected (Fig. 3b). In line with an HIF-independent upregulation of p27 mRNA, the hypoxic p27 level was not changed by PHD2, the main regulator of HIF, knockdown (Fig. 3b and c). Moreover, neither HIF-1α nor HIF-2α knockdown could revert the effect of PHD3 depletion on p27 expression (Fig. 3c and d). In line with this, 786-O cells that do not express functional HIF-1α show growth arrest under PHD3 depletion (Fig. 1). The data demonstrates that the PHD3-mediated p27 upregulation is neither transcriptional nor HIF-dependent once under hypoxia, although p27 may be transcriptionally upregulated by hypoxia from low normoxic levels [6].

In addition to HIF-dependency, we studied the role of hydroxylase activity on p27 expression using hydroxylase inhibitors DMOG and CoCl_2_. If the increase in p27 expression would be directly regulated by hydroxylase-dependent activity one would expect to see increased p27 levels at relative short time points. However, no effect on p27 expression was detected either in normoxia or hypoxia up to 8 h of DMOG exposure in HeLa (Additional file 1: Figure S2A) or 786–0 cells (Additional file 1: Figure S2B). Likewise, in HeLa cells CoCl_2_ did not increase p27 level as compared to hypoxia (Additional file 1: Figure S2C). An increase in p27 expression with DMOG was noted only after 24 h suggesting a transcriptional induction of p27 as described earlier [6, 38–40].

### PHD3 activates hypoxic degradation of p27

**Fig. 4:**
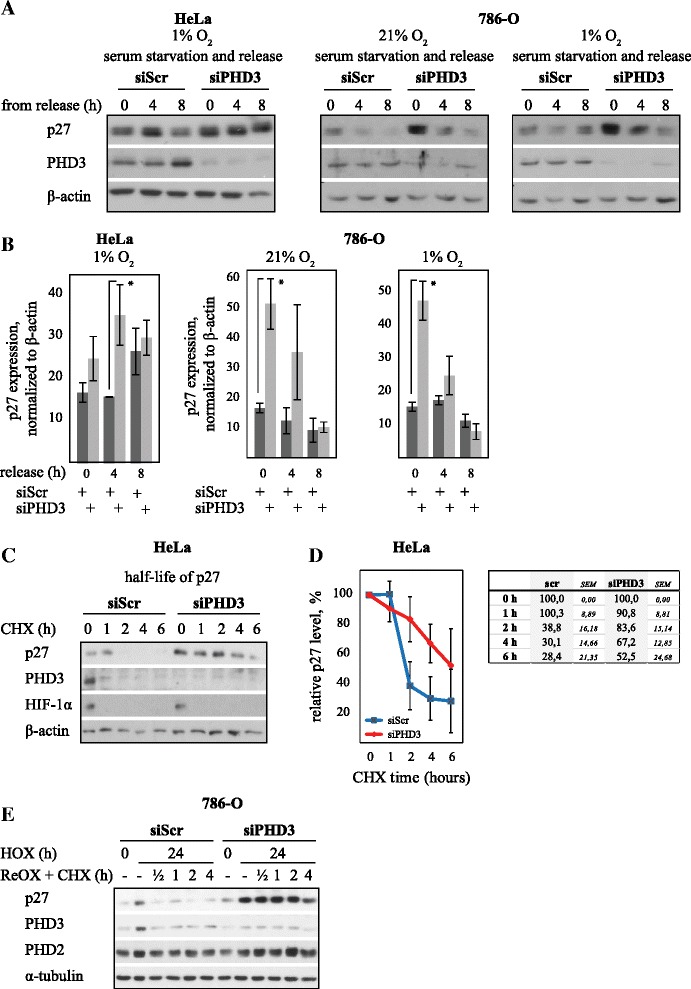
PHD3 depletion stabilizes hypoxic p27 expression by increasing p27 half-life. **a** Cell cycle arrest at G0 and subsequent release shows an increase of p27 expression in siPHD3 exposed cells. **b** Quantification for p27 expression under PHD3 depletion at indicated time points after cell cycle release in HeLa and 786-O cells. Asterisk indicates significant difference (*p* < 0,05; *n* = 3). **c** Cell cycle arrest at G0 and inhibition of protein synthesis with cycloheximide indicate increased p27 stability in PHD3 depleted HeLa cells. **d** Quantification of p27 expression using siPHD3 or control at indicated time points. Four independent experiments (± SEM) are shown (*p* < 0,05; *n* = 4). **e** Analysis of p27 stability in 786–0 cells by cycloheximide chase during reoxygenation after 24 h hypoxia demonstrates markedly increased half-life of p27 upon PHD3 depletion

To further explore the dynamics of siPHD3-mediated p27 induction siRNA-treated cells were synchronized with either serum starvation to G0 or with aphidicolin to S-phase. The expression of p27 was followed after cell cycle release up to eight hours (Fig. 4a and Additional file 1: Figure S3). Impairment in p27 degradation by PHD3 depletion was visible after both G0 and S-phase block but was most prominent after G0 phase block. After release from G0 arrest p27 expression started to decline steadily in control cells. However, in PHD3 depleted HeLa cells the decline was delayed and quantification of p27 level at different time points demonstrated a marked impairment of p27 decay (Fig. 4a and b). In 786-O cells the difference in serum starved cells was even more significant although p27 was decayed faster as compared to HeLa cells (Fig. 4a and b). As the delayed reduction in p27 expression was detected not only in serum starved but also in aphidicolin treated cells, and as we have previously shown the increase in p27 expression in unsynchronized cells, we concluded that the elevation of p27 was not a consequence of delay in cell cycle re-entry [24].

The difference on the impairment of p27 decay by PHD3 reduction was also seen after S-phase arrest and subsequent cell cycle re-entry (Additional file 1: Figure S3A). Hypoxic HeLa cells exposed to siPHD3 had almost complete block in p27 degradation at early time points up to six hours as compared to control following S-phase arrest (Additional file 1: Figure S3B). The data implied that PHD3 enhances p27 degradation at G0 to S phases under hypoxia.

In order to verify that the siPHD3-mediated delay in the disappearance of p27 is due to the disruption in post-translational degradation, cycloheximide (CHX) chase was performed. HeLa cells were synchronized either at G0 (Fig. 4c and d) or S phases (Additional file 1: Figure S3C and D), exposed to hypoxia and p27 stability was followed for six hours after protein translation block. In G0 the p27 levels are high in control cells and further elevated by siPHD3 at early time points. As expected, in the control cells the p27 levels begun to decline rapidly after cycloheximide induced translational block. In PHD3 deplete cells however, the p27 degradation was markedly attenuated at least up to six hours (Fig. 4c and d). At six hours after cycloheximide exposure the p27 expression was markedly elevated in PHD3 depleted cells as compared to control cells arrested at G0 (Fig. 4c). In line with previous results performed at S phase, the increased p27 stability by siPHD3 was even more pronounced although the overall levels of p27 in S phase are lower (Additional file 1: Figure S3C). Again, quantification of p27 expression up to six hours demonstrated that hypoxic cells arrested at S-phase and exposed to siPHD3 have almost complete block in p27 degradation (Additional file 1: Figure S3D). At six hours post-cycloheximide block the p27 expression was almost five-fold in PHD3 deplete cells compared to control. We further exposed 786–0 cells to 24-h hypoxia followed by translational block and reoxygenation (in order to further suppress p27 production) up to 4 h. In line with the previous data, p27 decay was fast in control cells but markedly delayed by PHD3 depletion (Fig. 4e). The data demonstrated that PHD3 is required for normal degradation of p27 under hypoxia. Noticeably, p27 expression being secondary to PHD3-induced cell cycle changes was ruled out as p27 half-life was measured after both G0 and S phase arrest.

### PHD3 depletion induced phosphorylation at serine 10 is required for increased hypoxic p27 stability

**Fig. 5:**
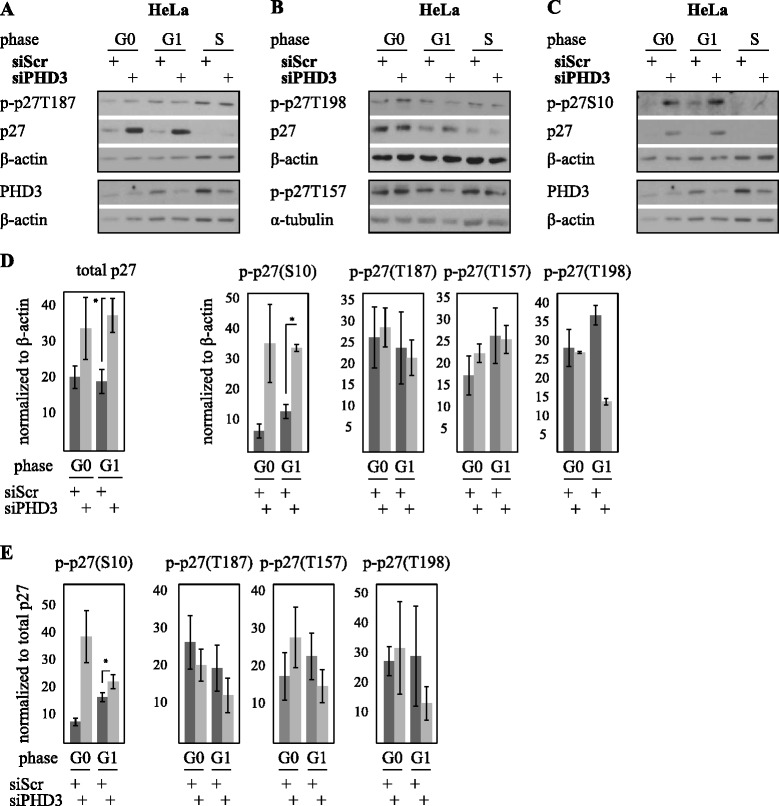
PHD3 depletion increases S10 phosphorylation of p27 in hypoxia. **a** Western blot analysis of p27 expression in cells arrested at different cell cycle phases and exposed to PHD3 or control siRNA. PHD3 depletion has no effect on T187 phosphorylation of p27 at any cell cycle phase. **b** PHD3 depletion has little effect on p27 phosphorylated on T157 and T198. **c** S10 phosphorylated form of p27 is strongly induced in PHD3 depleted cells. **d** Quantification of total and indicated p27 phosphoproteins from four independent experiments normalized to β-actin. Out of the phosphoproteins the effect on the expression of p27S10 is most prominent. Asterisk indicates significant difference (*p* < 0,005; *n* = 4) (**e**) Quantification of indicated p27 phosphoproteins from four independent experiments normalized to total p27 expression. The effect on the expression of p27S10 is most prominent and comparable to the total p27 expression. Asterisk indicates significant difference (*p* < 0,05; *n* = 4). Only minor effect on T157 and T198 are seen

**Fig. 6:**
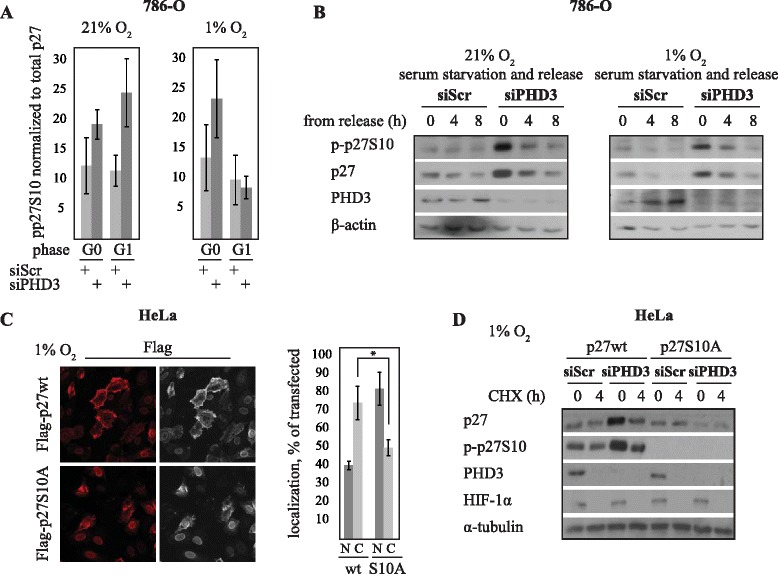
p27S10 phosphorylation is required for PHD3 depletion induced increase in p27 half-life. **a** Quantification of p27S10 phosphorylation from three independent experiments in 786-O cells. S10 phosphorylation demonstrated 2-fold increase at 6 h after cell cycle release in PHD3 depleted cells. **b** Serum starved 786-O cells were released from G0 block and S10 phosphorylation was monitored at the indicated timepoints under PHD3 depletion. PHD3 depletion had a prominent effect on S10 phosphorylation of p27. **c** Verification of subcellular localization of p27 plasmids. Immunofluorescence staining of Flag-p27wt / Flag-p27S10A transfected HeLa cells after 24 h hypoxia. As expected, p27wt is localized more into cytoplasmic than nuclear compartment. p27S10A is more localized into nucleus than into cytoplasm. Quantification of the subcellular localization from three optical fields (40x) (N; nuclear, C; cytoplasmic). **d** Flag-p27wt and S10A phosphorylation-deficient mutant (p27S10A) were transfected into siRNA-exposed HeLa cells. After 24 h of hypoxia CHX chase was performed. p27wt demonstrates strongly increased stability in siPHD3 exposed cells whereas p27S10A did not show any increased stability

PHD3 is known to regulate HIF ubiquitylation and degradation. However, we did not detect any PHD3-specific effect of proteasomal inhibition on p27 (Additional file 1: Figure S4), changes in p27 ubiquitylation (Additional file 1: Figure S4) nor was the p27 regulating ubiquitin ligase Skp2 expression altered by siPHD3 (Additional file 1: Figure S5). Moreover, no marked changes in p27 subcellular localization were detected (Additional file 1: Figure S6). Since the accumulation and functional activity of p27 is determined by phosphorylation and besides direct hydroxylation PHD3 has been implied to regulate protein phosphorylation [41–43], we therefore explored the phosphorylation of p27 at four known phosphorylation sites at different cell cycle phases under PHD3 depletion. Phosphorylation of threonine 187 (T187) was not affected by PHD3 depletion at any cell cycle phase (Fig. 5a, d and e). In PHD3-depleted cells there was only a minor effect on p27 phosphorylation on threonines 157 (T157) and 198 (T198), seen at G0 (Fig. 5b, d and e). In striking contrast, the phosphorylation of serine 10 (S10) was markedly increased by PHD3 depletion both in HeLa and 786-O cells (Figs. 5c-e, 6a and b). Quantification of the total expression level (normalized to β-actin) of each phosphorylation site illustrated prominently phosphorylated S10, which was comparable to the stabilization by siPHD3 seen on total p27. T157 showed no change, and T187 and T198 either no change or slight decrease in PHD3-dependent phosphorylation (Fig. 5d). Next the expression of the phosphorylated forms of p27 were normalized to total p27 levels. Again, S10 was the only phosphorylation site demonstrating strongly elevated phosphorylation by PHD3-depletion (Fig. 5e). This was seen both in hypoxic HeLa cells (Fig. 5e) and 786-O cells (Fig. 6a) at both G0 and G1 phases.

To further study the effect of PHD3 on S10 phosphorylation of p27, cells were synchronized at G0 and phosphorylated p27S10 expression was determined at different time points up to 8 h after serum starvation release. In line with the total p27 level, PHD3 depletion strongly attenuated the p27S10 reduction in 786–0 cells (Fig. 6b). The effect of PHD3 knockdown was seen at least up to four hours after cell cycle release as compared to control.

Finally, we used forced expression of a flag-tagged phosphorylation-deficient mutant (p27S10A) to study the effect of PHD3 on S10 phosphorylation. First the threshold level (Additional file 1: Figure S6A) for wild type p27 (p27wt) and S10-deficient mutant (p27S10A) expression as well as the normal localization by immunofluorescence staining (Fig. 6c) were determined. Mutated p27S10A is not detected by the phosphoserine specific antibody as expected as the phosphorylation site is removed and the overexpressed p27 exceeds the endogenous expression, thus on western blots mainly the exogenous p27 is detected. [16, 44]. The degradation of the S10-deficient mutant (p27S10A) was compared to that of wild type p27 (p27wt) in PHD3-depleted and cycloheximide exposed cells (Fig 6d). The data demonstrated that siPHD3 is not able to elevate the evel of p27 when S10 phosphorylation site is removed. siPHD3 attenuated the degradation of p27wt but the degradation of p27S10A remained unaffected or slightly decreased by siPHD3 (Fig. 6d). The data confirmed that PHD3 depletion increases the stability of p27 by affecting the phosphorylation of S10.

## Discussion

Hypoxia is a major factor in a number of physiological and pathological conditions. Hypoxia can cause cell cycle arrest of many cell types at the G1 phase and two distinct oxygen-dependent restriction points operating both in HIF-dependent and –independent manner have been described. Cancer cells, which commonly face hypoxia, are at least partially able to overcome the arrest in order to proliferate. Often they even exploit hypoxia to develop more aggressive features. At least three cyclin-dependent inhibitors including p27, p21 and p16 are upregulated by hypoxia but their role in the hypoxic cell cycle arrest and hypoxic cancer cell proliferation has been enigmatic [5–7]. Elevated p27 levels in hypoxia have been reported by several groups [4, 6, 24, 45–48]. Also increased cdk2-p27 interaction in diverse cell types under hypoxia in G1 or G1/S transition have been reported [4, 46, 47]. Moreover, using MEFs with p27−/− and knockdown, Gardner et al. [6], reported that p27 is required for hypoxic G1 arrest. However, Green et al. [5] using immortalized MEFs, reported that p27 is not necessary for the onset of hypoxic cell cycle arrest in S-phase and one report did not see a correlation between p27 levels and G1 arrest [49]. Therefore, the role of p27 in hypoxic cell cycle regulation remains somewhat controversial although several publications argue for a crucial role for p27 in hypoxic G1 arrest.

Here we have described one mechanism as to how cancer cells may overcome the hypoxia-induced cell cycle arrest at G1/S by affecting p27 levels. We have demonstrated that PHD3 drives the carcinoma cell cycle by regulating the stability of p27 in conditions with high PHD3 level. The reduced amount of p27 allows cell cycle to proceed from G1 to S even under hypoxia. This occurs by PHD3 driven decrease in the phosphorylation of p27 at serine 10 (S10). Phosphorylation of S10 has been previously shown to stabilize p27 effectively also *in vivo* and suggested to present the most stabile form of p27 [14, 15, 50]. We have further shown that the reduced hypoxic survival of PHD3-depleted cells is mediated by S10 phosphorylation-induced high expression of p27.

The regulation of p27 expression is complex and is known to be dependent on the cell cycle phase with high level at G0 and strongly reduced level at the S-phase. We ruled out an indirect effect of cell cycle phase on our results by arresting cells at either G0 or S-phase and studying the effect of PHD3 on p27 expression. PHD3 depletion strongly suppressed p27 decay under hypoxia even when the cell cycle was halted indicating that PHD3 does not convey its effects to p27 destabilization indirectly through affecting other steps in cell cycle regulation (Fig. 4 and Additional file 1: Figure S2). In support of a direct effect on p27, p27 knockdown rescued the PHD3 depletion induced hypoxic cell cycle block (Fig. 2).

Phosphorylation of p27 at T187 and S10 has been reported to regulate p27 stability. Hypoxic PHD3 depletion increased only S10 phosphorylation indicating that T187 phosphorylation or SCF-Skp2 mediated proteasomal degradation of p27 are not involved in the hypoxic PHD3-mediated p27 regulation. Moreover, although the effect of PHD3 on p27 expression was clearly not transcriptional or HIF-dependent we could not see any marked effect of PHD3 knockdown on proteasomal degradation or ubiquitylation of p27 (Additional file 1: Figure S3), suggesting that under hypoxia PHD3-mediated p27 destabilization is regulated independently of proteasomal degradation. This was further supported by the fact that Skp2 expression did not change upon PHD3 reduction (Additional file 1: Figure S4) and that the expression of p21, another target of Skp2, was unchanged (Fig. 1b) (reviewed in [51]). In normoxia S10 phosphorylation is known to affect the subcellular localization. We could not detect any major influence of PHD3 on p27 cytoplasmic localization (Additional file 1: Figure S5), suggesting that under hypoxia the change in S10 phosphorylation is not necessarily followed by p27 translocation. However, the effect of PHD3 depletion on p27 degradation was prominent. This is in line with previous studies showing that S10 phosphorylation stabilizes p27 [14, 15]. Our data using forced expression of increasing plasmid amount of p27wt and p27S10A to study cell growth in hypoxia showed that cell amount correlated with the increasing p27 level and was independent on S10 (Additional file 1: Figure S6B and C). This was in line with previous studies reporting that there is no marked difference between the wild type and S10-deficient mutant neither in proliferation nor cell cycle progression in normoxia [52–54]. Accordingly, cell cycle analysis at two distinct time points under hypoxia showed no difference on cell cycle progression between p27wt and p27S10A (Additional file 1: Figure S6D and E). The overexpression analyses argue that the effects of PHD3 on cell cycle progression are mediated through elevation of total p27 level rather than changes in, e.g., p27 localization.

PHD3 has been reported to influence protein phosphorylation such as IKKβ [41], FAK [42] and Akt [43]. The exact mechanisms as to how a proline hydroxylase regulates phosphorylation remain obscure but are likely to be indirect. Human kinase interacting stathmin (hKIS) is known to phosphorylate p27 on S10 and thereby is a possible target for PHD3 [55]. However, we did not detect any changes in hKIS expression under PHD3 depletion (not shown). As Akt and ERK2 have been reported to phosphorylate p27 on S10, PHD3 could potentially regulate the upstream effectors of p27 [14, 56]. In support of this PHD3 has been reported to diminish Akt phosphorylation and to impair glucose metabolism [43]. There is a clear difference in the decay of p27 between HeLa and renal cell carcinoma cells (786-O) (Fig. 4). This is of interest as 786-O cell line has been shown to have high activity of Akt and therefore strong relocalization of p27 into cytoplasmic compartment directed by T157 phosphorylation [57]. Yet another plausible mechanism is enhanced dephosphorylation of S10 by PHD3. Such enhanced hypoxia-induced phosphatase activity has been reported for example for Smad3 phosphorylation [58].

PHD3 has been shown to have multiple other targets besides HIF-1α and many of these are involved in cellular adaptation and signaling pathways enabling cell survival (reviewed in [59]). The reported functions of PHD3 include regulation of apoptosis in normoxia [23, 25, 26, 60, 61], effects on cellular metabolism through pyruvate kinase M2 (PKM2) [62, 63], regulation of NF-κB signaling [41, 64, 65] together with the regulation of cell cycle progression [24]. These multiple functions of PHD3 are context specific as they vary depending on the tissue oxygenation level, pVHL mutation status as well as cell type. However, they imply that PHD3 is a crucial hypoxia-responsive factor at the crossroad of several survival signaling pathways. Finally, it is noteworthy that besides PHD3 also PHD1 is known to support cell cycle [35]. There are however, clear differences between PHD1 and −3 in the cell cycle regulation. First, the effect of PHD1 depletion was found to be strictly hydroxylase-dependent and the effects on cell cycle progression were studied in normal oxygen pressure while the effect of PHD3 depletion is seen mainly under hypoxic conditions or when PHD3 is upregulated by the lack of functional pVHL and seems to be hydroxylase activity-independent. In addition, PHD1 affects mitosis whereas PHD3 operates during G1 phase. Importantly however, together these findings imply crucial role of the oxygen sensing machinery in controlling cell cycle progression.

## Materials and methods

### Cell culture, synchronization and cycloheximide chase

HeLa cells were obtained from ATCC (Rockville, MD, USA) and cultured in Dulbecco’s Modified Eagle Medium (DMEM, Sigma-Aldrich) supplied with 10 % fetal calf serum (FCS), L-glutamine and antibiotics (penicillin and streptomycin). Cells were cultured in 5 % CO_2_ in 37 °C. For hypoxic experiments the cells were cultured in 1 % oxygen in a hypoxic workstation (Invivo_2,_ Ruskinn Technology). For synchronizing the cells into G0 10 % FCS DMEM was replaced with 0,1 % FCS DMEM 24–48 h after indicated transfection. Cells were serum starved for 48 h. For S phase block aphidicolin (Sigma-Aldrich) was used at 1 μg/ml for 24 h. Samples for western blotting and flow cytometry were collected at indicated timepoints after cell cycle release.

For cycloheximide chase cells were transfected with siRNAs followed by cell synchronization. After 24 h of hypoxic exposure cells were washed once and supplied with fresh hypoxia-balanced DMEM and CHX (Sigma-Aldrich).

For reoxygenation experiments cells were grown in hypoxia for 24 h after which CHX was added. Cells were reoxygenized by exposing them on normal oxygen pressure and samples were collected at the indicated timepoints.

### Transfections, antibodies and reagents

For siRNA transfections two stranded oligonucleotides were used at final concentration of 10–20 nM. Transfections were performed using Oligofectamine™ or Lipofectamine® RNAiMAX (Invitrogen) according to manufacturer’s protocol. The siRNAs (MWG Biotech AG) used were: non-target (siScr) 5′-CCUACAUCCCGAUCGAUGAUG(dTdT)-3′, siEPAS1/HIF-2α 5′-GCGACAGCUGGAGUAUGAAUU(dTdT)-3′, siHIF-1α 5′-AACUAACUGGACACAGUGUGU(dTdT)-3′, siPHD1 5′-ACAUUGCUGCAUGGUAGAA(dTdT)-3′, siPHD2 5′-GACGAAAGCCAUGGUUGCUUG (dTdT)-3′, siPHD3 5′-GUCUAAGGCAAUGGUGGCUUG (dTdT)-3′ and sip27 5′-AAGCACACUUGUAGGAUAA (dTdT)-3′. For adenoviral shRNA delivery HeLa cells were transduced with either control (Ad-shScr) 5′-GACACGCGACTTGTACCACTTCAAGAGAGTGGTACAAGTCGCGTGTCTTTTTTACGCGT-3′ or with PHD3-targeting shRNA (Ad-shPHD3) 5′- CCGGCACCTGCATCTACTATCTGAACTCGAGTTCAGATAGTAGATGCAGGTGTTTTT-3′ (Vector BioLabs).

Plasmids for p27 overexpression studies were kindly provided by Dr. K. I. Nakayama (Kyushu University, Japan). Transfections were performed using Fugene® HD (Promega) according to manufacturer’s protocol.

Antibodies used were: PHD3 (NB100-139, Novus Biologicals), PHD2 (NB100-137, Novus Biologicals), PHD1 (NB100-310, Novus Biologicals), Flag (F3165, Sigma-Aldrich), HIF-1α (610959, BD Transduction Laboratories), EPAS1/HIF-2α (NB100-122, Novus Biologicals), p16 (554079, BD Pharmingen/sc-468, Santa Cruz Biotechnology Inc.), p21 (sc-397, Santa Cruz Biotechnology Inc.), p27 (sc-528, Santa Cruz Biotechnology Inc.), p-p27(S10) (sc-12939-R, Santa Cruz Biotechnology Inc.), p-p27(T157) (AF1555, R&D Systems), p-p27(T187) (sc-16324, Santa Cruz Biotechnology Inc.), p-p27(T198) (AF3994, R&D Systems), Skp2 (sc-7164, Santa Cruz Biotechnology Inc.) and β-actin (Ac-74, Sigma-Aldrich). For protein degradation studies proteasome inhibitor MG132 (Sigma-Aldrich) was used at 10 μM final concentration and cycloheximide (CHX, Sigma-Aldrich) at 10 μg/ml. PHD inhibitor DMOG was used at 1 mM and CoCl_2_ at 200 μM final concentration.

### RT-PCR, protein analysis and flow cytometry

For real time PCR mRNA was extracted using NucleoSpin RNA II kit (Macherey-Nagel, Düren, Germany) and reverse transcription using M-MuLV RNase H-reverse transcriptase (Finnzymes, ThermoFisher, Waltham, MA, USA) according to the manufacturer’s protocol. RT–PCR reactions were run using Applied Biosystems 7900HT Fast Sequence Detection System and TaqMan Universal Master Mix II, no UNG (Applied Biosystems, Life Technologies, Carlsbad, CA, USA). Taqman primers (Oligomer) and probes (Roche, Universal ProbeLibrary) used are listed in Additional file 1: Table S1. mRNA expression was normalized against β-actin.

For protein expression analysis cells were harvested in SDS-Triton lysis buffer. Protein concentration was measured using Bio-Rad DC Protein assay and protein detection using Pierce ECL Western blotting substrate (Thermo Scientific).

For flow cytometry cells were incubated 24–48 h to reach 50–60 % confluence and synchronized as described, fixed with 70 % ethanol and stained with propidium iodide. Cell cycle analysis was performed using flow cytometer (BD FACSCalibur, BD Biosciences) and BD CellQuest™ Pro software.

### Imaging and immunocytochemistry

For cell counting the cell nuclei cells were fixed with fresh 4 % paraformaldehyde and stained with the nuclear stain Hoechst 33342 (Invitrogen). Optical fields of cells were imaged with Zeiss Lumar V12 fluorescence stereo microscope (Carl Zeiss) and the number of nuclei per optical field was calculated using ImageJ software (NIH, USA). Experiments were done as parallel treatments and each experiment was repeated at least three times.

### Data analysis and statistics

Western blots were quantified using Image J or BioRad ChemiDoc MP and Image Lab software for band analysis. Intensities were normalized to β-actin. Data is presented as mean ± SEM. The statistical significance was evaluated using a 2-tailed, paired Student’s *t*-test. Differences were considered statistically significant at *p* < 0,05.

## Additional file

Additional file 1:
**Supplementary data.** (PDF 288 kb)
