# Supplementary material for: Hypoxia inducible prolyl hydroxylase PHD3 maintains carcinoma cell growth by decreasing the stability of p27
Source: Mol Cancer. 2015 Jul 30;14:143. doi: 10.1186/s12943-015-0410-5 (PMC4520080; doi:10.1186/s12943-015-0410-5)
Supplement: Additional file 1: — Supplementary data. (PDF 288 kb) [file 12943_2015_410_MOESM1_ESM.pdf]

Supplement figure S1

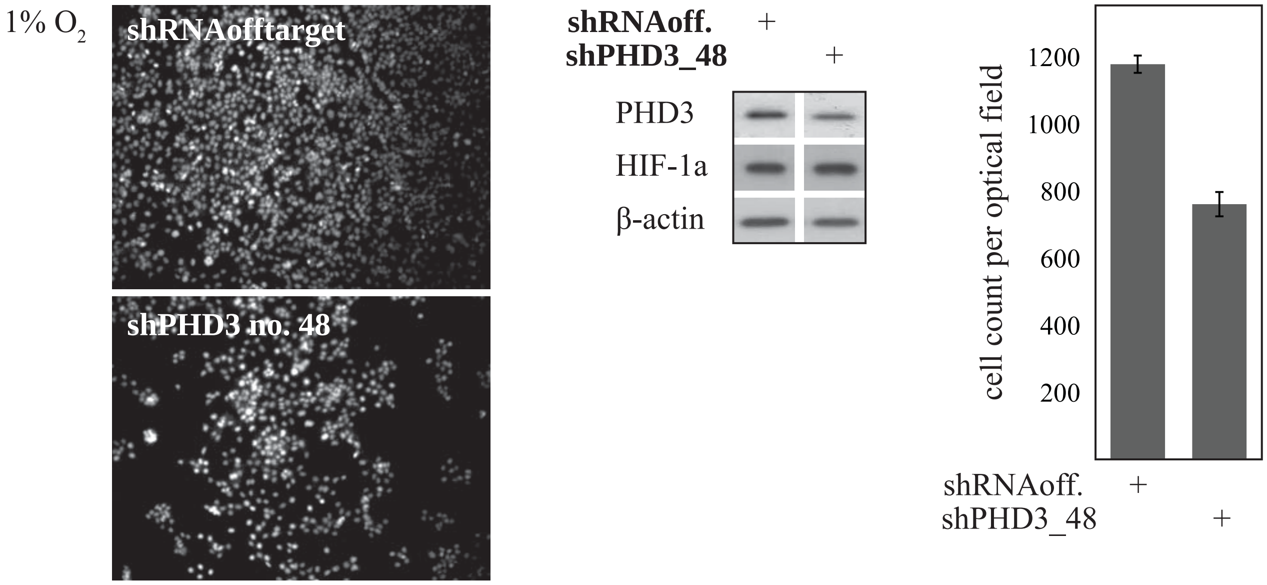

Figure S1: The effect of PHD3 delpetion by RNAi is sequence-independent. shRNA targeted against PHD3 was used (MISSION shRNA, clone NM_022073.3-764s21c1, Sigma-Aldrich) with a corresponding offtarget control shRNA. The effect on cell count in prolonged hypoxia (48 h, 1 % O_2) was the same as with the siRNA with different sequence.

Supplement figure S2

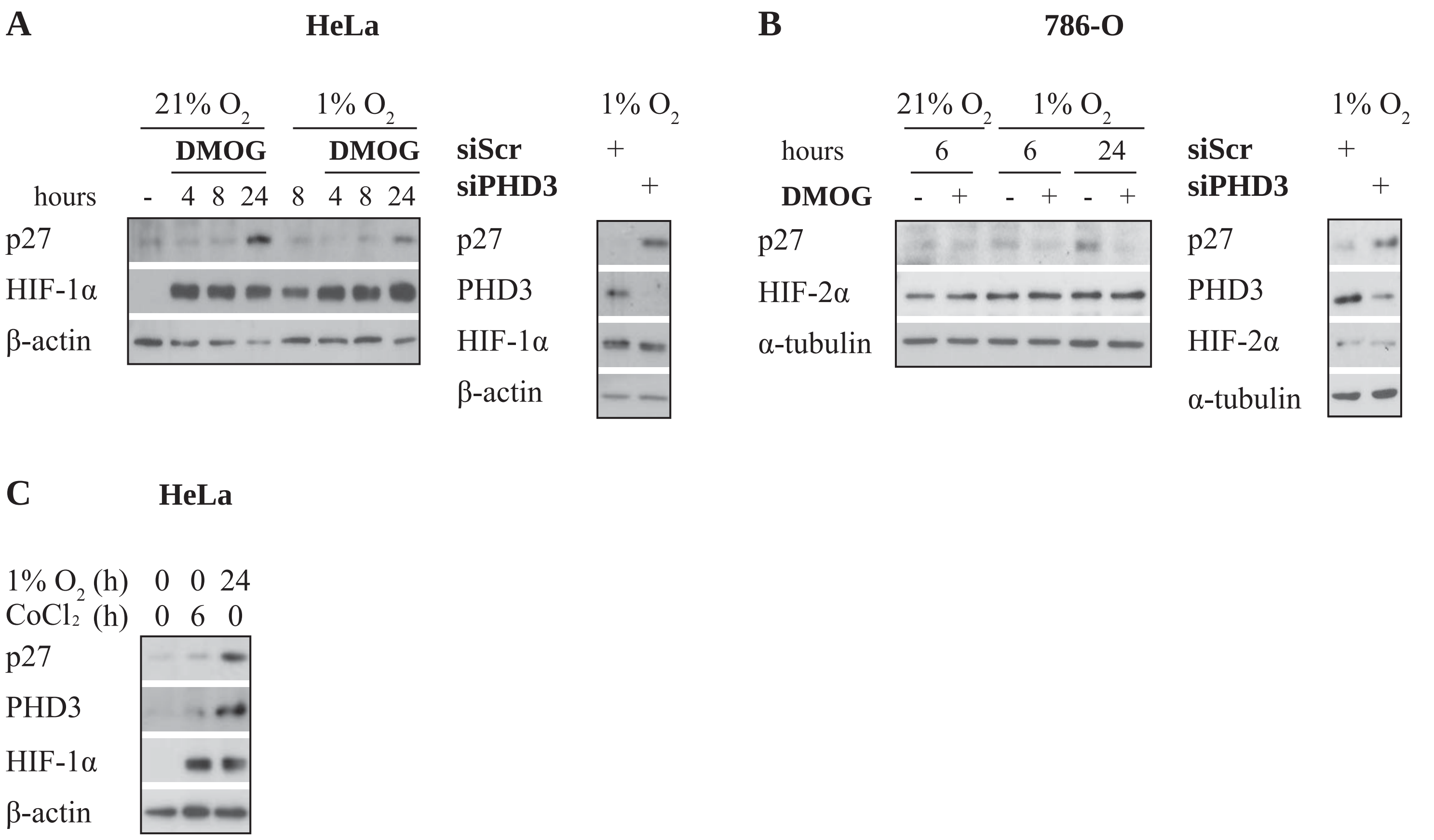

Figure S2: PHD3-mediated p27 regulation does not require hydroxylase activity.

Supplement figure S3

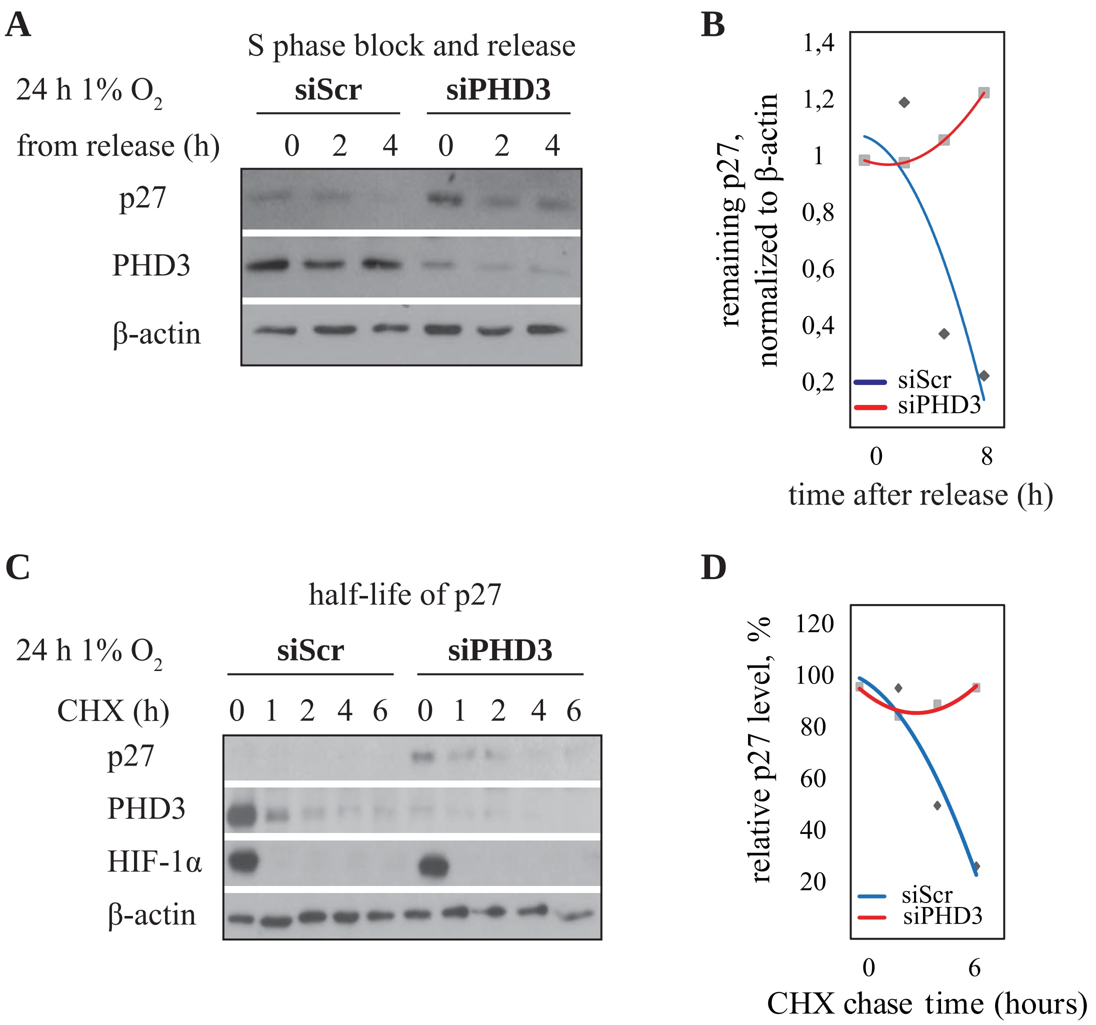

Figure S3: Aphidicolin-mediated cell cycle block in early S phase show increased p27 stability in PHD3 depleted cells. (A) Cell cycle release from S phase block does not initiate the degradation of p27 in PHD3 depleted cells in contrast to control cells. (B) Quantification and histogram of p27 expression at indicated time points after S phase block. Three independent experiments ±SEM are shown. (C) Cell cycle arrest and inhibition of protein synthesis with cycloheximide at early S phase indicate increased p27 stability in PHD3 depleted cells. ((D) Quantification of p27 half-life of three independent experiments shows an almost total blockage of p27 degradation upon PHD3 depletion.

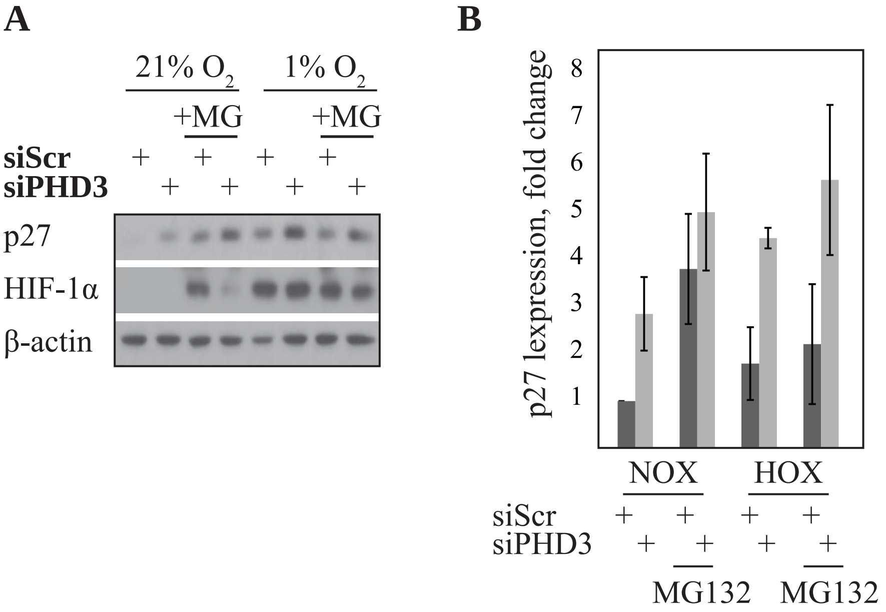

Figure S4: PHD3 mediated p27 regulation is not proteasomal. (A) p27 is induced in normoxia but not in hypoxia with proteasome inhibitor MG132. siPHD3 depletion increases p27 expression whether the proteasomal degradation is inhibited or not. (B) Quantification of three independent experiments ±SEM are shown. (C) Ubiquitination of p27 is unaffected in siPHD3 cells. HeLa cells were transfected with HA-tagged ubiquitin and HA-IP was performed in hypoxia. MG132-treated cells served as a positive control.

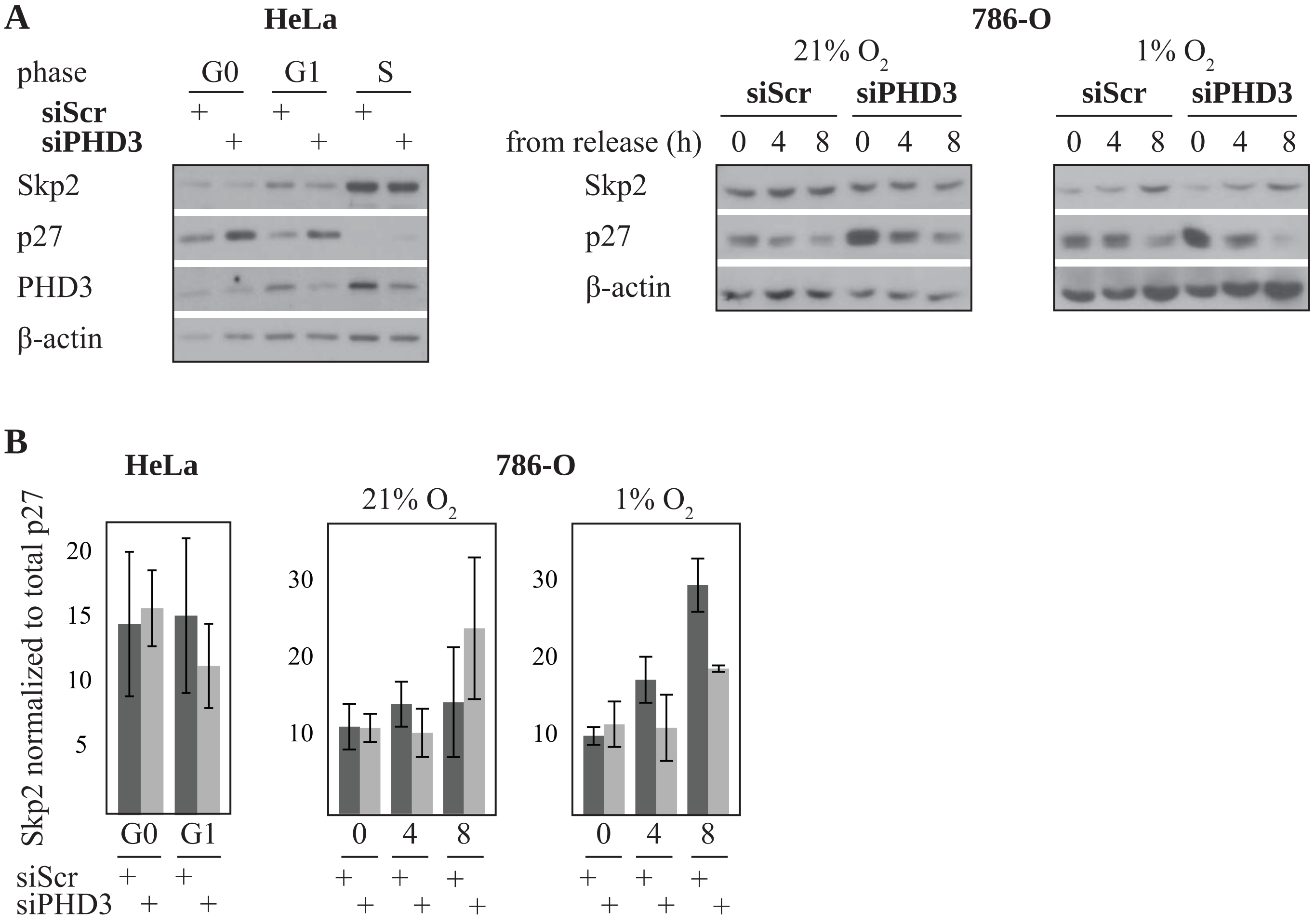

Figure S5: The main degradation pathway of p27 is mediated by T187 phosphorylation targeted by SCF-Skp2 ubiquitin ligase. (A) Skp2 expression was monitored in HeLa and 786-O cells at indicated cell cycle timepoints. (B) Quantification of Skp2 expression. Three independent experiments ±SEM are shown.

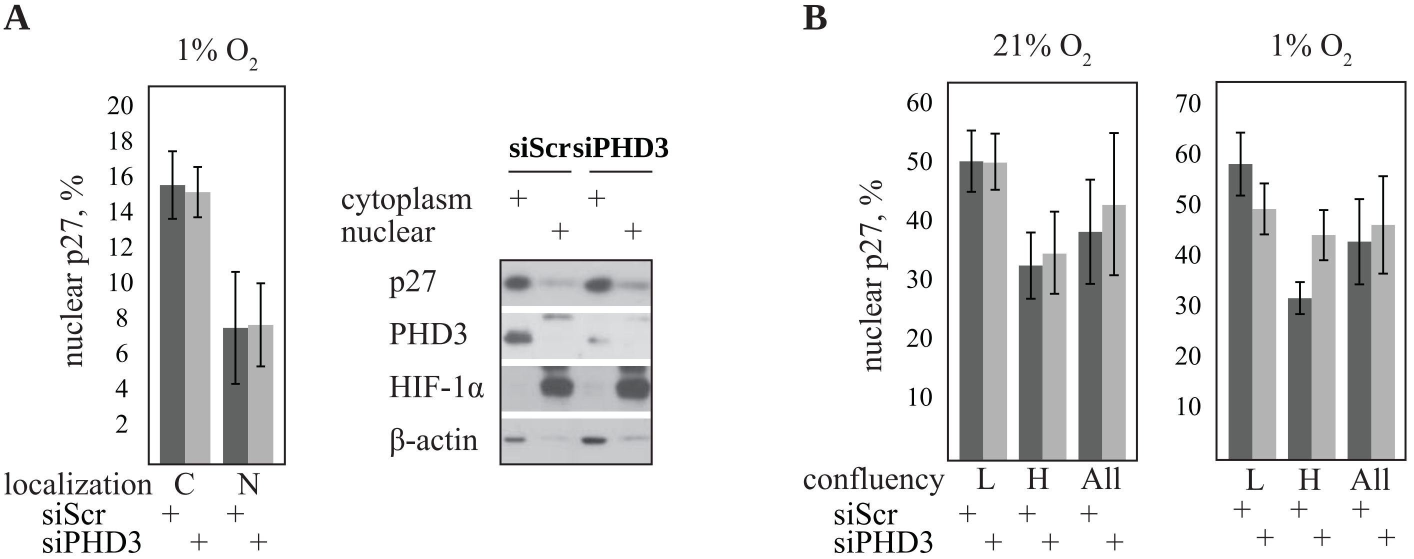

Figure S6: PHD3 depletion traps p27 in the nucleus in high confluency/during contact inhibition. (A) Cell fractionation and following western blot analysis show now change in the localization of p27 in PHD3 depleted cells. Quantification of three independent experiments. N; nuclear, C; cytoplasmic. (B) Cytological staining of p27 in HeLa cells after 24 hours hypoxia. Quantification of the subcellular localization from five optical fields per three independent experiments (40x). Confluency was determined ocularly: L; <80 % confluent, H; ≥80 % confluent.

Supplement Figure S7

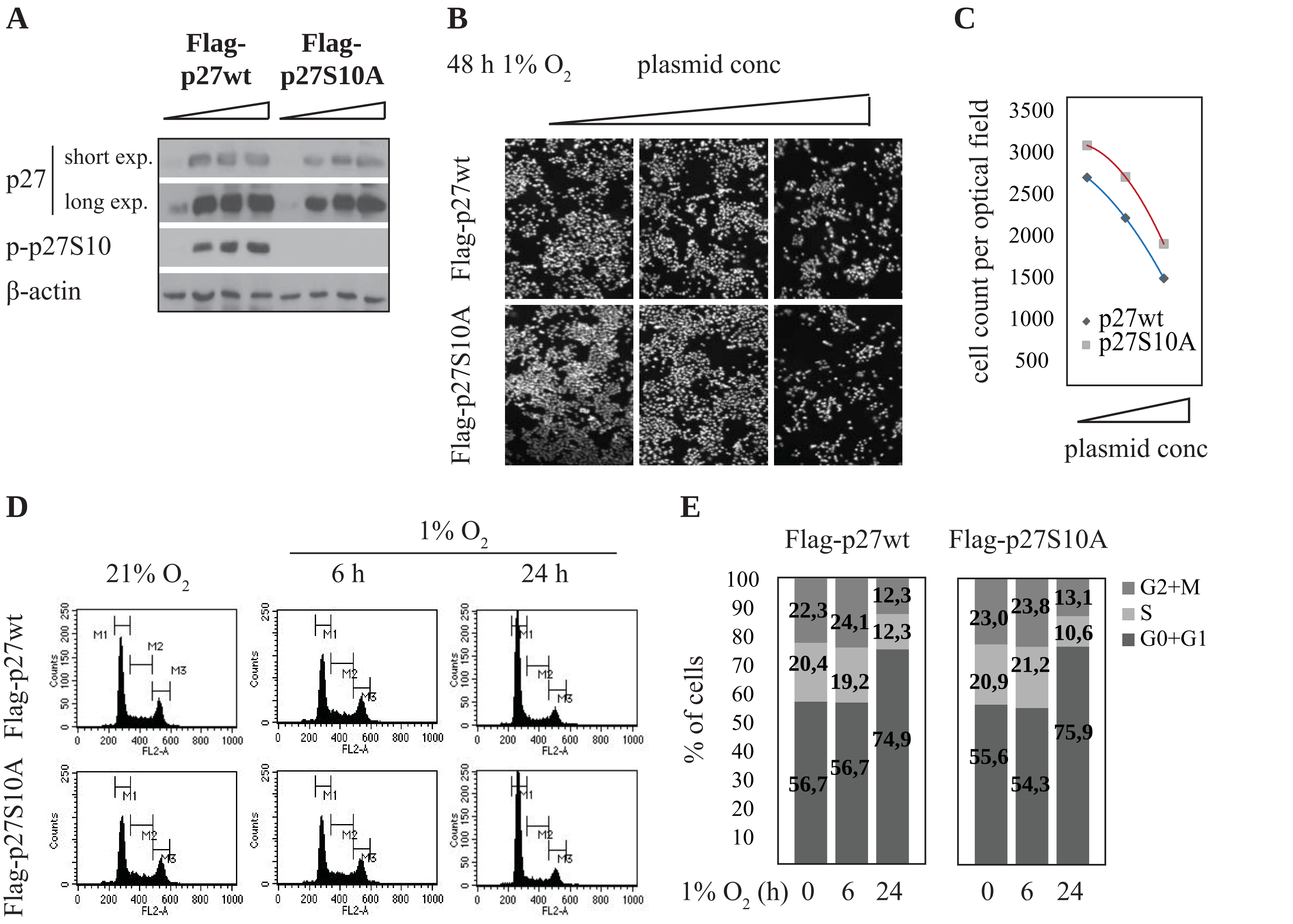

Figure S7: The effect of p27 on hypoxic cell cycle depends on the expression level. (A) Western blot analysis of titration of Flag-p27wt and Flag-p27S10A expression by increasing plasmid concentration. (B) The inhibitory effect of p27 on cell cycle is concentration-dependent and depends mainly on p27 expression level only, not serine phosphorylation. HeLa cells were transfected with increasing amounts of p27wt and S10A mutant and exposed to hypoxia for 48 hours. Cells were visualized by Hoechst staining. (C) Quantification of cell amount from four independent experiments ±SEM are shown. No significant difference in cell amount between p27wt and p27S10A transfected cells were detected. (D) The effect of ectopically expressed p27 on cell cycle is not dependent on S10 phosphorylation of p27. HeLa cells transfected with the indicated p27 plasmids and exposed to hypoxia followed by FACS analysis. (E) Quantification of cell cycle phases from a representative FACS analysis.

Supplemental Experimental Procedures

qRT-PCR

Table S1: Primers and probes used in qRT-PCR

Target	Primer left	Primer right	Probe (Universal ProbeLibrary, Roche)
phd3	atcgacaggctggtcctcta	gatagcaagccaccattgc	**#61** (cat. no. 04688597001)
p27	tttgacttgcatgaagagaagc	agctgtctctgaaagggacatt	**#60** (cat. no. 04688589001)
glut1	ggttgtgccatactcatgacc	cagataggacatccagggtagc	**#67** (cat. no. 04688660001)
epas1/hif-2α	catctacaaccctcgcaacc	tggagaacaccacgtcattc	**#45** (cat. no. 04688058001)

Cellular fractionation

Cell fractionation was performed 48-72 hours after siRNA transfection. Cells were lysed in cold Buffer 1 (10 mM Hepes pH 7,9; 10 mM KCl, 100 µM EDTA, 100 µM EGTA; 0,1 % NP-40; 1,5 mM $MgCl_2$ supplemented with 20 mM β-glycerolphosphate, 25 mM NaF, 500 µM PMSF, 1 mM DTT and complete protein inhibitor) and cytoplasmic proteins were collected from the supernatant. The precipitate was resuspended in cold lysis buffer 2 (20 mM Hepes pH 7,9; 400 mM NaCl, 250 µM EGTA; 1,5 mM $MgCl_2$, 10 % glycerol supplemented with 20 mM β-glycerolphosphate, 25 mM NaF, 500 µM PMSF, 500 µM DTT and complete protein inhibitor) and nuclear proteins were collected from the supernatant. Antibodies against β-actin and HIF-1α were used as markers for cytoplasmic and nuclear fractions, respectively.

Immunoprecipitation

For immunoprecipitation experiments (IP) HA-tagged ubiquitin was used (HA-Ub). Cells were seeded on 10 cm plates at plating density of $2{,}5x10^5$ cells/ml. Plasmid transfections with HA-Ub were performed 24 hours later. Primary selection for transfected cells with G418 was started 24 hours after transfections and continued for 48 hours. siRNA transfections were performed as described. Hypoxic exposure for 24 hours was started 24 hours after siRNA transfections. For positive control MG132 was added to the cells 4 hours before collecting the samples. To collect the samples cells were first washed twice and then collected in cold PBS by scraping. Sample tubes

were centrifuged in +4ºC for 5 min, 1500 rpm. Supernatant was discarded and cell pellet was resuspended in ice cold lysis buffer (50 mM Tris-HCl, pH 7,5; 150 mM NaCl, 1 mM EDTA; 0,2 % TX-100 supplemented with 20 mM β-glycerophosphate, 100 μM Na_3VO_4; 0,5 mM PMSF, 1 mM DTT, 5 μM MG132, 10 mM N-ethylmaleimide and complete protein inhibitor). Lysed cells were centrifuged in +4ºC for 5 min, 13 200 rpm. Supernatant was collected into two separate tubes and 5 % input sample was taken. Cellular lysate was incubated with HA antibody under rotation in +4ºC o/n, after which 25 μl of magnetic protein A beads (Abnova) was added to the reaction mixture and incubated for 2 hours in +4ºC. Washes were performed on magnetic separation rack: supernatant was removed and beads were washed three times with cold lysis buffer with 5 min rotation. Immunoprecipitated proteins were boiled in TXLB sample buffer for 10 min and run on SDS-PAGE.

Imaging and immunocytochemistry

The cells were fixed with fresh 4 % paraformaldehyde (PFA) or with PTEMF fixing solution (100 mM PIPES pH 6.8, 10 mM EGTA, 1 mM $MgCl_2$, 0,2 % Triton X-100 and 4 % formaldehyde) and stained with the nuclear stain and with Flag antibody. Optical fields of cells were imaged with Zeiss LSM510 META. Experiments were done as parallel treatments and each experiment was repeated at least three times.
